# PSM Peptides From Community-Associated Methicillin-Resistant *Staphylococcus aureus* Impair the Adaptive Immune Response via Modulation of Dendritic Cell Subsets *in vivo*

**DOI:** 10.3389/fimmu.2019.00995

**Published:** 2019-05-10

**Authors:** Jennifer R. Richardson, Nicole S. Armbruster, Manina Günter, Michelle Biljecki, Juliane Klenk, Simon Heumos, Stella E. Autenrieth

**Affiliations:** ^1^Department of Internal Medicine II, University of Tübingen, Tübingen, Germany; ^2^Quantitative Biology Center, University of Tübingen, Tübingen, Germany

**Keywords:** dendritic cells, *Staphylococcus aureus*, phenol-soluble modulins, T cells, mouse infection, adaptive immunity

## Abstract

Dendritic cells (DCs) are key players of the immune system and thus a target for immune evasion by pathogens. We recently showed that the virulence factors phenol-soluble-modulins (PSMs) produced by community-associated methicillin-resistant *Staphylococcus aureus* (CA-MRSA) strains induce tolerogenic DCs upon Toll-like receptor activation via the p38-CREB-IL-10 pathway *in vitro*. Here, we addressed the hypothesis that *S. aureus* PSMs disturb the adaptive immune response via modulation of DC subsets *in vivo*. Using a systemic mouse infection model we found that *S. aureus* reduced the numbers of splenic DC subsets, mainly CD4^+^ and CD8^+^ DCs independently of PSM secretion. *S. aureus* infection induced upregulation of the C-C motif chemokine receptor 7 (CCR7) on the surface of all DC subsets, on CD4^+^ DCs in a PSM-dependent manner, together with increased expression of MHCII, CD86, CD80, CD40, and the co-inhibitory molecule PD-L2, with only minor effects of PSMs. Moreover, PSMs increased IL-10 production in the spleen and impaired TNF production by CD4^+^ DCs. Besides, *S. aureus* PSMs reduced the number of CD4^+^ T cells in the spleen, whereas CD4^+^CD25^+^Foxp3^+^ regulatory T cells (T_regs_) were increased. In contrast, Th1 and Th17 priming and IFN-γ production by CD8^+^ T cells were impaired by *S. aureus* PSMs. Thus, PSMs from highly virulent *S. aureus* strains modulate the adaptive immune response in the direction of tolerance by affecting DC functions.

## Introduction

The Gram-positive bacterium *Staphylococcus aureus* is a frequent member of the human microbiota, but it is also the most common cause of bloodstream infections (bacteremia) and the leading cause of more than 50% of skin and soft-tissue infections worldwide (1, 2). Especially the community-associated methicillin-resistant *S. aureus* (CA-MRSA) strains, such as USA300, are linked to morbidity and mortality due to their high virulence, not only in immunocompromised patients but also in healthy individuals (3). CA-MRSA strains efficiently evade the host's innate and adaptive immune system by the expression of a great variety of virulence factors, like Panton-Valentine Leukocidin, α-toxin, and phenol-soluble modulin (PSM) peptides, which are highly secreted by these strains (1, 3–5). PSMs contain five α-peptides (δ-toxin and PSMα1-4) and two β-peptides (PSMβ1-2), which all share an amphipathic α-helical structure, thereby acting as biological detergents. Thus, PSMs are regarded as a new class of *Staphylococcal* leukocidins (5, 6). PSMs first attract innate immune cells like neutrophils, macrophages, and dendritic cells (DCs) by binding to the formyl peptide receptor 2 (FPR2) (5, 7–9). Subsequently, α-type PSMs were shown to lyse neutrophils, monocytes and erythrocytes, but not DCs, via membrane perturbation, thereby evading the innate immune response (6, 7, 9–11). Moreover, *S. aureus* infection impairs proliferation of B cells and T cells, which prevents the establishment of protective immune responses (4).

DCs are specialized antigen presenting cells that link the innate and adaptive immunity and are the only cells able to prime naïve T cells thereby inducing a primary immune response and maintaining self-tolerance (12). Immature DCs are specialized to internalize antigens and get activated by inflammatory signals recognized by germ-line encoded pattern recognition receptors (PRRs), like Toll-like receptors (TLRs) (13). As a result, DCs reduce antigen uptake by downregulating endocytosis, yet enhance antigen processing and presentation, and upregulate the C-C motif chemokine receptor 7 (CCR7), co-stimulatory- and MHC class II (MHC II) molecules, necessary for homing into the draining lymph node and for efficient T-cell priming (12, 14). TLRs respond to exogenous microbial products, which results in the activation of the downstream signaling pathways that leads to the expression of cytokines, chemokines, and interferons (15, 16). The pro-inflammatory cytokines, e.g., TNF-α, IL-6, and IL-12 recruit other immune cells for pathogen clearance and induce T helper cell differentiation (17). In contrast, anti-inflammatory cytokines, like IL-10 possess important immunoregulatory functions by, e.g., inhibiting IL-12 production, which promotes regulatory T-cell (T_reg_) development (18). In lymphoid organs, DCs are subdivided into CD4^+^ DCs, CD8^+^ DCs, and CD4^−^CD8^−^ DCs (double negative (DN) DCs), which have distinct functions. CD4^+^ DCs predominantly activate CD4^+^ T cells via MHC class II presentation, whereas CD8^+^ DCs are specialized for CD8^+^ T cell priming via cross-presentation (12, 19). Systemic *S. aureus* infections of DC-depleted mice demonstrated that these cells are essential for the survival of and bacterial killing within the host (20).

Previously, we showed that PSMs modulate the maturation and cytokine production of human and mouse DCs. As a result, these PSM-treated tolerogenic DCs (tDCs) promote priming of CD4^+^CD25^+^Foxp3^+^ T_regs_ whereas T helper 1 (Th1) priming was impaired (9, 11, 21, 22), which indicates another immune evasion mechanism of *S. aureus* PSMs. However, the *in vivo* relevance is missing. To address the hypothesis that *S. aureus* PSMs disturb the adaptive immune response via modulation of DC subsets *in vivo*, wild type (WT) and FPR2-deficient mice were intravenously infected with the *S. aureus* USA300 WT strain or PSM mutant strains and the effects of PSMs on DC numbers and functions and in consequence their ability to prime T cells was assessed.

## Materials and Methods

### Mice

Animal experiments were performed in strict accordance with the German regulations of the Society for Laboratory Animal Science (GV-SOLAS) and the European Health Law of the Federation of Laboratory Animal Science Associations (FELASA). The protocol was approved by the Regierungspräsidium Tübingen (Permit Numbers: IZ2/12, M1/14). Female C57BL/6J Rj mice were purchased from Janvier (St. Berthevin Cedex, France). FPR2^−/−^ (23), and Foxp3-eGFP mice (24) with a genetic C57BL/6 background were bred in the animal facilities of the University Hospital Tübingen. All mice were held under specific pathogen-free conditions, were provided with food and water *ad libitum* and used for experiments between 6 and 12 weeks of age.

### Bacteria

*Staphylococcus aureus* USA300 WT and the PSM deletion mutant strains *S. aureus* USA300 Δαβδ, USA300 Δα, USA300 Δβ, and USA300 Δδ (5) were kindly provided by Prof. Andreas Peschel, University of Tübingen. All strains were handled according to biosafety level two regulations from trained personnel.

### Infection of Mice

C57BL/6J, FPR2^−/−^ and Foxp3-eGFP mice were infected with 3 × 10^7^ CFU of *S. aureus* USA300 WT or the PSM deletion mutant strains *S. aureus* USA300 Δαβδ, USA300 Δα, USA300 Δβ, or USA300 Δδ in 200 μl PBS into the tail vein for the indicated time.

The bacterial load in the spleen and the kidney was assessed by plating serial dilutions on Tryptic soy agar or Columbia agar with sheep blood plates (Oxoid). Mice injected with 200 μl PBS into the tail vein served as controls.

### Purification

For flow cytometry analysis 1/3 of the spleen was used. For DC analysis a single cell suspension was prepared from the spleen using collagenase as described previously (25). For the experiments where only T cells were analyzed the spleens were pressed through a 70 μm strainer with the pistil of a 2 ml syringe in 3 ml PBS to create a single cell suspension. Erythrocytes were lysed with a lysis buffer containing 150 mM NH_4_Cl, 10 mM KHCO_3_, and 2 mM NaEDTA.

The remaining 2/3 of the spleen was used to prepare a homogenate for analyzing cytokine production. For this, the spleen was incubated for 10 min on ice in 1 ml ice-cold PBS with 0.1% Igepal CA-630 (Sigma-Aldrich) and for inhibition of proteolytic activity complete protease inhibitor cocktail tablets (Roche) were added according to the manufacturer's instructions. After the incubation, the spleen was pressed through a 40 μm strainer with the pistil of a 2 ml syringe and the cell suspension was pelleted and frozen at −20°C until cytokine analysis. For analyzing cytokine production in the plasma, blood was drawn and centrifuged for 10 min at 3,500 rpm at 4°C. The plasma was taken and frozen at −80°C until cytokine analysis.

For *in vitro* restimulation experiments, 2.5 × 10^6^ splenocytes from PBS-treated or infected mice were harvested and cultured in 400 μl of RPMI 1,640 (Biochrom) supplemented with 5% FBS (Sigma-Aldrich), 2 mM glutamine (Life Technologies), 100 U/ml penicillin/streptomycin (Life Technologies), 1x non-essential amino acids (Biochrom), 10 mM Hepes (Life Technologies), 1 mM Na-pyruvate (Biochrom), 0.004% β-mercaptoethanol (Roth) in a 48-well plate in the presence of PMA (Sigma-Aldrich) and ionomycin (Sigma-Aldrich) at 37°C. Two hours later Brefeldin A (BioLegend) was added for another 4 h, followed by flow cytometry staining (described below).

### Flow Cytometry Staining

For DC or T-cell analysis in the spleen single cell suspensions were incubated with Fc-block to prevent unspecific binding (cell culture supernatants of the hybridoma cell line 2.4G2) for 10 min at 4°C. After that, extracellular staining was performed for 20 min at 4°C using the antibodies listed in Table S1. If CCR7 was included in the panel, cells were stained with the CCR7 antibody for 15 min at 37°C before the staining with the other extracellular antibodies. Dead cells were excluded using either 7-amino actinomycin D (Biomol) after the extracellular staining or using Zombie Aqua/NIR (BioLegend) before extracellular staining and Fc-block according to the manufacturer's instructions. Next cells were fixed and permeabilized (Foxp3 Staining Buffer Set, eBioscience) and stained intracellularly with antibodies listed in Table S1. The cells were washed with PBS with 1% FBS and 2 mM EDTA (Sigma-Aldrich) and acquired using a Canto-II or LSRFortessa flow cytometer (BD Biosciences) with DIVA software (BD Biosciences). Data analysis was performed using FlowJo software V.10.5 (Tree Star).

### Cytokine Production

Fifteen microliter of the spleen homogenates or plasma were analyzed for cytokine production by performing a bead-based immunoassay in a 96-well plate [LEGENDplex mouse inflammation panel (13-Plex)] according to the manufacturer's instructions, using the Lyric flow cytometer with an autosampler (BD Bioscience).

### Statistical Analysis

Statistical analysis was performed with the GraphPad Prism 7 software (GraphPad, San Diego, CA). When comparing only two groups within the same timepoint, an unpaired Student's *T*-Test was used for significance analysis. When three or more different groups were compared, a one-way ANOVA with Tukey's *post-hoc* test was conducted in order to assess the significance. The differences were considered as statistically significant if *p* < 0.05 (^*^), *p* < 0.005 (^**^), *p* < 0.001 (^***^), or *p* < 0.0001 (^****^). The absence of an asterix automatically means that no significance was reported. For the details of the number of independent experiments see Table S2.

## Results

### Reduced Numbers of Splenic DC Subsets Upon *S. aureus* Infection

To investigate the impact of *S. aureus* infection and especially PSMs on DC subsets *in vivo* C57BL/6J mice were either treated with PBS or infected with 3 × 10^7^ CFU *S. aureus* USA300 WT or the PSM deletion mutant strain *S. aureus* USA300 Δαβδ for 3, 6, 12, 24, or 72 h. Only small differences regarding signs of infection or the bacterial load in the spleen and kidney were observed between mice infected with *S. aureus* USA300 WT or the USA300 Δαβδ mutant strain over time. However, the numbers of splenocytes were significantly reduced in a PSM-dependent manner 72 h post infection (pi) (Figure S1).

**Figure 1:**
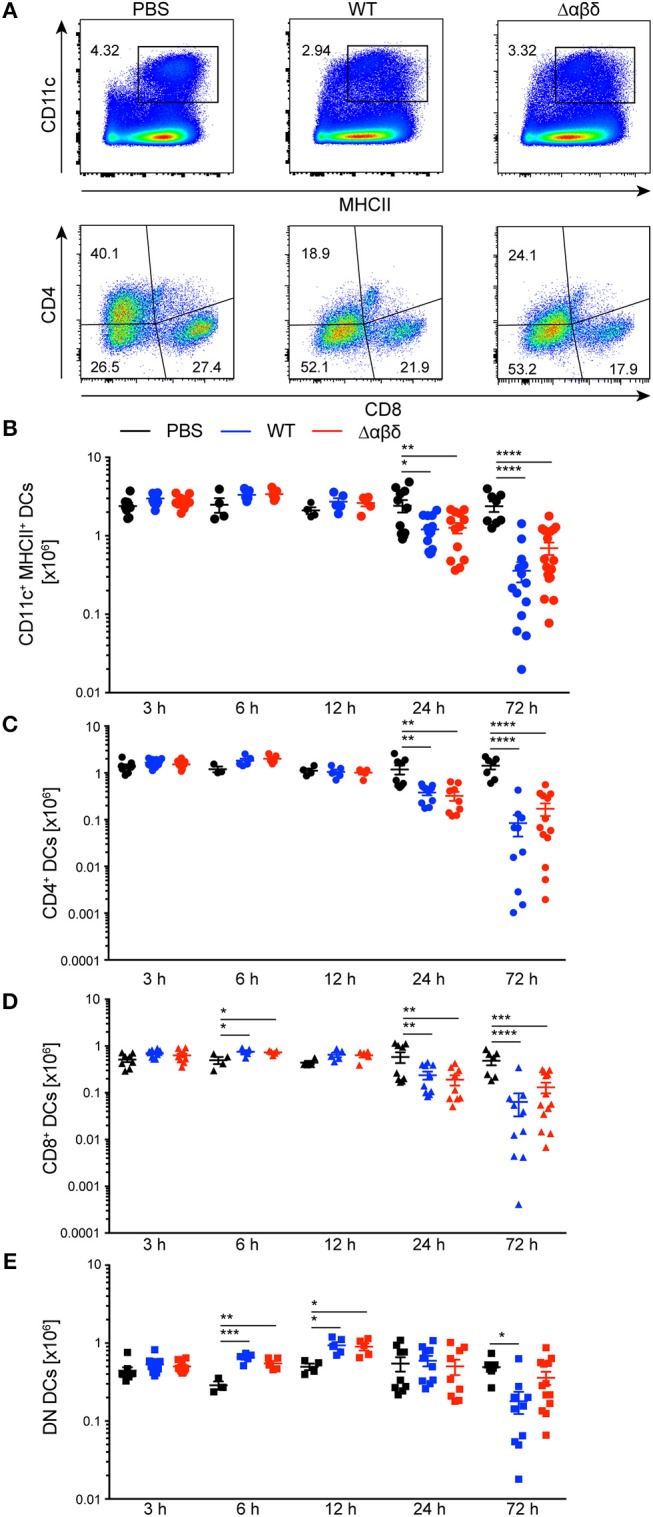
Reduced numbers of splenic DC subsets upon *S. aureus* infection. C57BL/6J WT mice were either treated with PBS or infected with *S. aureus* USA300 WT or the PSM-deficient *S. aureus* USA300 Δαβδ mutant strain for up to 3 days and splenocytes were analyzed for DC subsets by flow cytometry. **(A)** Pseudocolor plots show the frequency of classical DCs gated as singlets, leukocytes, living, lineage (CD19, NK1.1, Ter-119, GR-1, CD90.2)^−^, CD11c^hi^MHCII^+^ cells (upper panel) or the CD4^+^, CD8^+^, and DN DC subsets (lower panel) in the spleen of PBS-treated or infected mice. Graphical summary of the numbers of splenic CD11c^+^MHC^+^ DCs **(B)**, CD4^+^ **(C)**, CD8^+^ **(D)**, and DN DCs **(E)** over time. Every symbol represents one mouse; quantity of mice and experiments per condition and time is depicted in Table S2. The graphs represent the mean ± SEM with data pooled from multiple experiments [one-way analysis of variance (ANOVA) followed by Tukey's *post-hoc* test; ^*^p < 0.05, ^**^p < 0.01, ^***^p < 0.001, ^****^p < 0.0001].

DCs in the spleen were characterized as living lineage (CD19, NK1.1, Ter-119, Gr-1, CD90.2)^−^CD11c^hi^MHCII^+^ leukocytes and the DC subsets were further subdivided by their expression of CD4 and CD8 (Figure 1A, Figure S2). The variations between DC subset numbers from PBS-treated mice can be explained by circadian fluctuations throughout the daily cycle (26) (Figure 1). The number of DCs was significantly reduced at 24 h (PBS: 3.56 ± 0.92 × 10^6^; WT: 1.47 ± 0.43 × 10^6^, Δαβδ 1.75 ± 0.13 × 10^6^) and even more at 72 h pi with the *S. aureus* USA300 WT or the USA300 Δαβδ mutant strain compared to PBS treatment (PBS: 2.38 ± 1.04 × 10^6^; WT: 0.36 ± 0.39 × 10^6^, Δαβδ 0.69 ± 0.52 × 10^6^) [Figures 1A (upper panel),B]. Similar results were obtained for CD4^+^ and CD8^+^ DCs with significantly reduced cell numbers 24 and 72 h pi with *S. aureus* USA300 WT and Δαβδ, which was not observed for DN DCs (Figures 1C–E, Figure S3). Overall these data point toward a PSM-independent depletion of DC subsets in the spleen after infection with *S. aureus*, which is not based on a higher cell death rate (Figure S4).

### PSMs Enhance the *S. aureus* Infection-Induced DC Maturation

**Figure 2:**
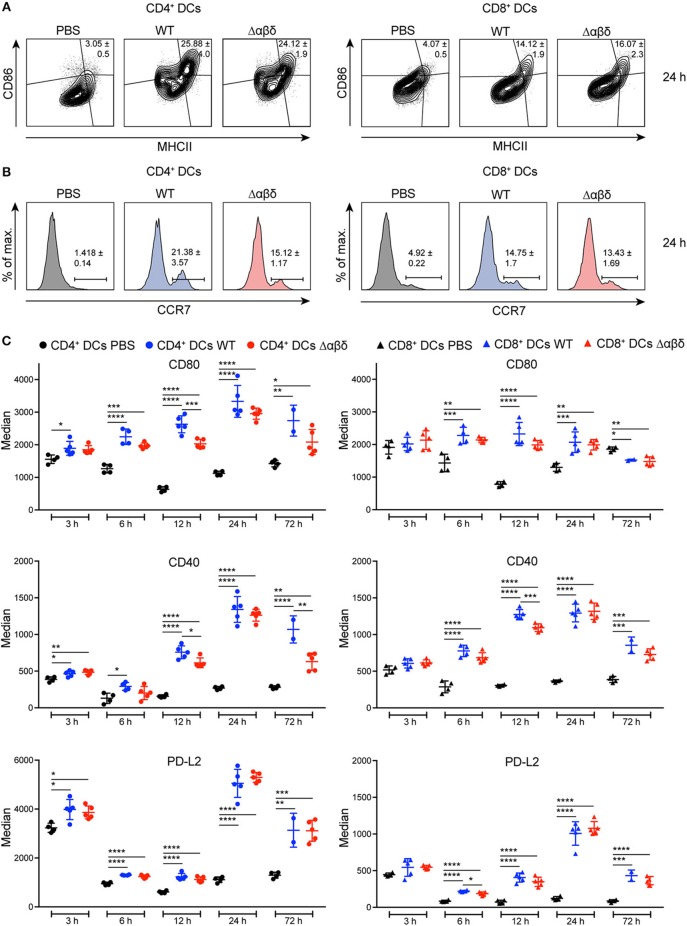
DC maturation upon *S. aureus* infection. C57BL/6J WT mice were either treated with PBS or infected with *S. aureus* USA300 WT or the PSM-deficient *S. aureus* USA300 Δαβδ mutant strain for up to 3 days and splenocytes were stained with CD11c, MHC class II, CCR7, CD80, CD86, CD40, and PD-L2 antibodies and analyzed by flow cytometry. Representative contour plots and histograms show the frequency of **(A)** MHC II^+^CD86^+^ cells and **(B)** CCR7^+^ cells, respectively, among CD4^+^ (left) and CD8^+^ (right) DCs in the spleen of PBS-treated or infected mice after 24 h. **(C)** Graphical summary of the median expression of CD80, CD40, and PD-L2 by CD4^+^ (left) and CD8^+^ (right) DCs in the spleen of PBS-treated or infected mice over time. Every symbol represents one mouse; quantity of mice and experiments per condition and time is depicted in Table S2. The graphs represent the mean ± SEM with data from one or pooled from 2 experiments (one-way ANOVA followed by Tukey's *post-hoc* test; ^*^p < 0.05, ^**^p < 0.01, ^***^p < 0.001, ^****^p < 0.0001).

DC maturation is characterized by the upregulation of MHC class II, co-stimulatory, co-inhibitory molecules, and the CCR7. Infection with the *S. aureus* USA300 WT or the *S. aureus* Δαβδ mutant strain induced the up-regulation of MHCII, CD86, CCR7, CD80, CD40, and PD-L2 on all DC subsets in the spleen starting 6 h pi compared to PBS-treated mice as analyzed by flow cytometry staining (Figure 2). The frequencies of CD86^+^MHC II^+^ DC subsets were increased by 3.5 to 8-fold 24 h pi with *S. aureus* USA300 WT or *S. aureus* Δαβδ mutant strain compared to PBS treatment (Figure 2A, Figures S5A,D–F). Similar results were observed for the frequencies of CCR7^+^ DC subsets with the tendency of even more CCR7^+^CD4^+^ DCs upon infection with the PSM expressing WT strain (Figure 2B, Figures S5B,D–F). The expression of the analyzed surface markers differed between the DC subsets upon infection, with the highest expression of CD80 and PD-L2 on the CD4^+^ DC subset (Figure 2C, Figure S5C). PSMs by trend enhanced the expression of the co-stimulatory molecules CD80 and CD40, but not of PD-L2, with the most prominent effects seen for CD8^+^ DCs at 12 h pi and for CD4^+^ DCs at 72 h pi (Figure 2C). These data show that all splenic DC subsets mature upon systemic *S. aureus* infections, with PSM peptides slightly enhancing this effect.

### *S. aureus* PSMs Enhance the Pro-inflammatory and Anti-inflammatory Cytokine Response Upon *in vivo* Infection

**Figure 3:**
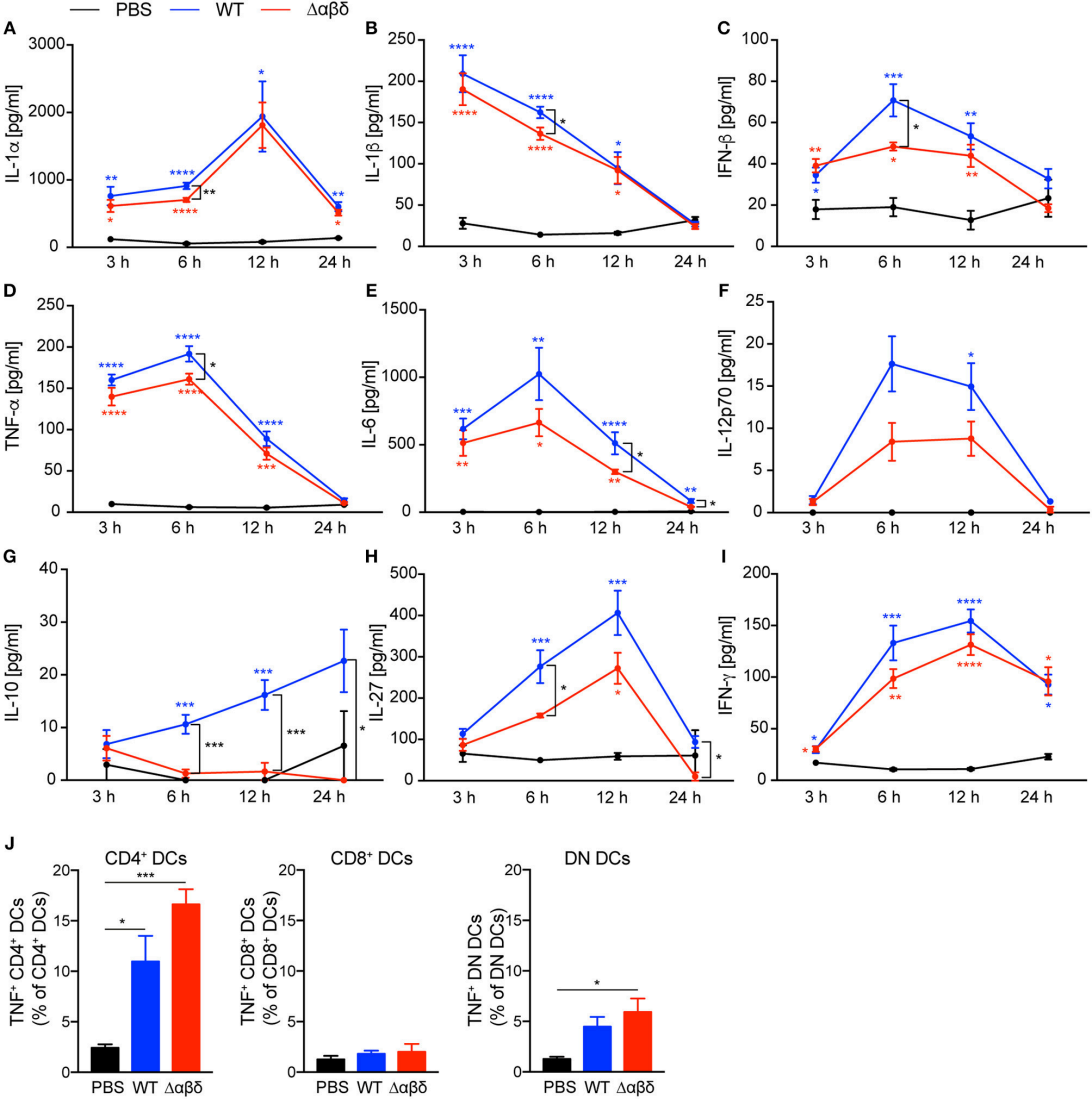
*S. aureus* PSMs modulate the pro-inflammatory and anti-inflammatory cytokine response upon *in vivo* infection. C57BL/6J WT mice were either treated with PBS or infected with *S. aureus* USA300 WT or the PSM-deficient *S. aureus* USA300 Δαβδ mutant strain for up to 24 h. At the indicated times spleen homogenates were analyzed for **(A)** IL-1α, **(B)** IL-1β, **(C)** IFN-β, **(D)** TNF-α, **(E)** IL-6, **(F)** IL-12p70, **(G)** IL-10, **(H)**, IL-27, and **(I)** IFN-γ by a multiplex bead array. The graphs represent the mean ± SEM with data from one or data pooled from multiple experiments; quantity of mice and experiments per condition and time is depicted in Table S2 (one-way ANOVA followed by Tukey's *post-hoc* test; ^*^p < 0.05, ^**^p < 0.01, ^***^p < 0.001, and ^****^p < 0.0001; blue ^*^ describe differences between PBS and *S. aureus* USA300 WT, red ^*^ describe differences between PBS and *S. aureus* USA300 Δαβδ, and black ^*^ describe differences between *S. aureus* USA300 WT and USA300 Δαβδ). **(J)** Graphical summary of the frequencies of TNF^+^ cells among DC subsets in the spleen of PBS-treated or infected mice (mean ± SEM of five mice from one experiment; one-way ANOVA followed by Tukey's *post-hoc* test; ^*^p < 0.05 or ^***^p < 0.001).

The encounter of bacterial products by PRRs expressed by DC subsets leads to cytokine secretion. We analyzed spleen homogenates (Figure 3) and blood plasma (Figure S6) for cytokine production from mice infected as described above. Infection of mice with *S. aureus*-induced an early inflammatory cytokine response with high levels of IL-1α, IL-1β, IFN-β, TNF-α, IL-6, IL-12p70, IL-27, and IFN-γ in the spleen detected for up to 12 h pi compared to PBS-treated mice (Figure 3). Besides, serum levels for MCP-1, TNF-α, and IL-6 were increased already 3 h pi with *S. aureus* compared to PBS-treated mice followed by IL-12 and IFN-γ 6 h pi (Figure S6), indicating an infection-induced proinflammatory response. By trend, the PSM secreting WT strain revealed higher production of pro-inflammatory cytokines compared to the *S. aureus* Δαβδ mutant strain. Of note, infection with the USA300 WT led to a significantly enhanced production of IL-10 at 6 to 24 h pi in the spleen, but the infection with the *S. aureus* Δαβδ mutant strain did not induce IL-10 secretion (Figure 3G), indicating a PSM-dependent anti-inflammatory response. The impact of PSMs on the cytokine profile in the blood plasma was less prominent, only showing increased IL-6 production upon infection with the *S. aureus* USA300 WT strain (Figure S6). In summary, PSMs enhance the pro- and anti-inflammatory immune response in the spleen during the first 12 h of infection.

Moreover, TNF-α production by all splenic DC subsets, detected by intracellular cytokine staining, was increased 24 h pi with *S. aureus* compared to PBS treatment with the tendency of even more TNF-α production by CD4^+^ and DN DC subsets of mice infected with the *S. aureus* Δαβδ mutant strain (Figure 3J, Figure S6F). These data suggest that PSMs affect TNF-α production by specific DC subsets.

### PSM Peptides Impair CD4^+^ T Cell and Promote Regulatory T-Cell Responses

**Figure 4:**
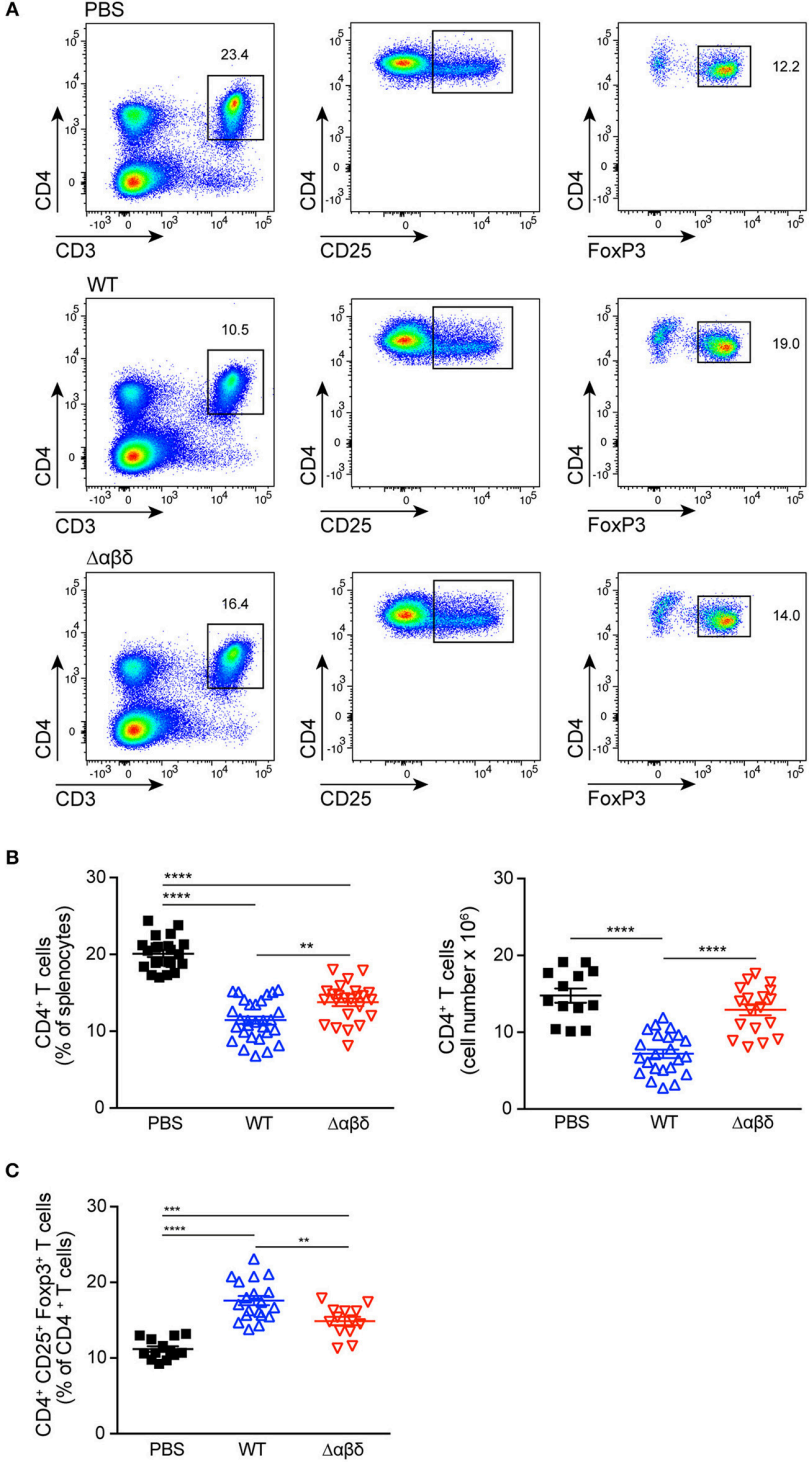
PSMs modulate CD4^+^ T cell responses. Foxp3-eGFP mice were either treated with PBS or infected with *S. aureus* USA300 WT or the PSM-deficient S. aureus USA300 Δαβδ mutant strain for 3 days and splenocytes were analyzed for CD4^+^ T cells by flow cytometry. **(A)** Pseudocolor plots show the frequencies of CD4^+^ and CD4^+^CD25^+^Foxp3^+^ T cells in the spleen of PBS-treated or infected mice. Graphical summary of the frequencies and cell numbers per organ of splenic CD4^+^ T cells **(B)** and the frequencies CD4^+^CD25^+^Foxp3^+^ T cells among CD4^+^ T cells **(C)**. Every symbol represents one mouse. The graphs represent the mean ± SEM with data pooled from multiple experiments; quantity of mice and experiments per condition and time is depicted in Table S2 (one-way ANOVA followed by Tukey's *post-hoc* test; ^**^p < 0.01, ^***^p < 0.001, and ^****^p < 0.0001).

DCs play a crucial role in the activation and polarization of CD4^+^ T cells (12). Therefore, CD4^+^ T-cell responses in the spleen were analyzed by flow cytometry upon infection of mice with *S. aureus* USA300 WT or USA300 Δαβδ mutant strain for 72 h or treatment with PBS. *S. aureus* infection led to significantly reduced frequencies (PBS: 20.1 ± 0.4%, WT: 11.4 ± 0.5%; Δαβδ mutant: 13.8 ± 0.5%) and numbers (PBS: 14.8 ± 0.9 × 10^6^, WT: 7.2 ± 0.6 × 10^6^; Δαβδ mutant: 12.9 ± 0.7 × 10^6^) of living CD3^+^CD4^+^ T cells in the spleen, which was partially PSM-dependent (Figures 4A,B). Similar findings were observed when mice deficient for the PSM receptor FPR2 were infected with *S. aureus* USA300 WT strain or the *S. aureus* USA300 Δαβδ mutant strain (Figure S7).

Using Foxp3-eGFP mice to accurately detect *in vivo* generated CD4^+^CD25^+^Foxp3^+^ T_regs_, the infection with the *S. aureus* USA300 WT strain led to a significantly increased frequency (PBS: 11.2 ± 0.4%, WT: 17.6 ± 0.6%; mutant: 14.9 ± 0.6%) of T_regs_ in the spleen compared to PBS-treated mice. This increase in T_reg_ frequency was partially PSM-dependent (Figures 4A,C), but independent of the FPR2 receptor signaling (Figure S7).

**Figure 5:**
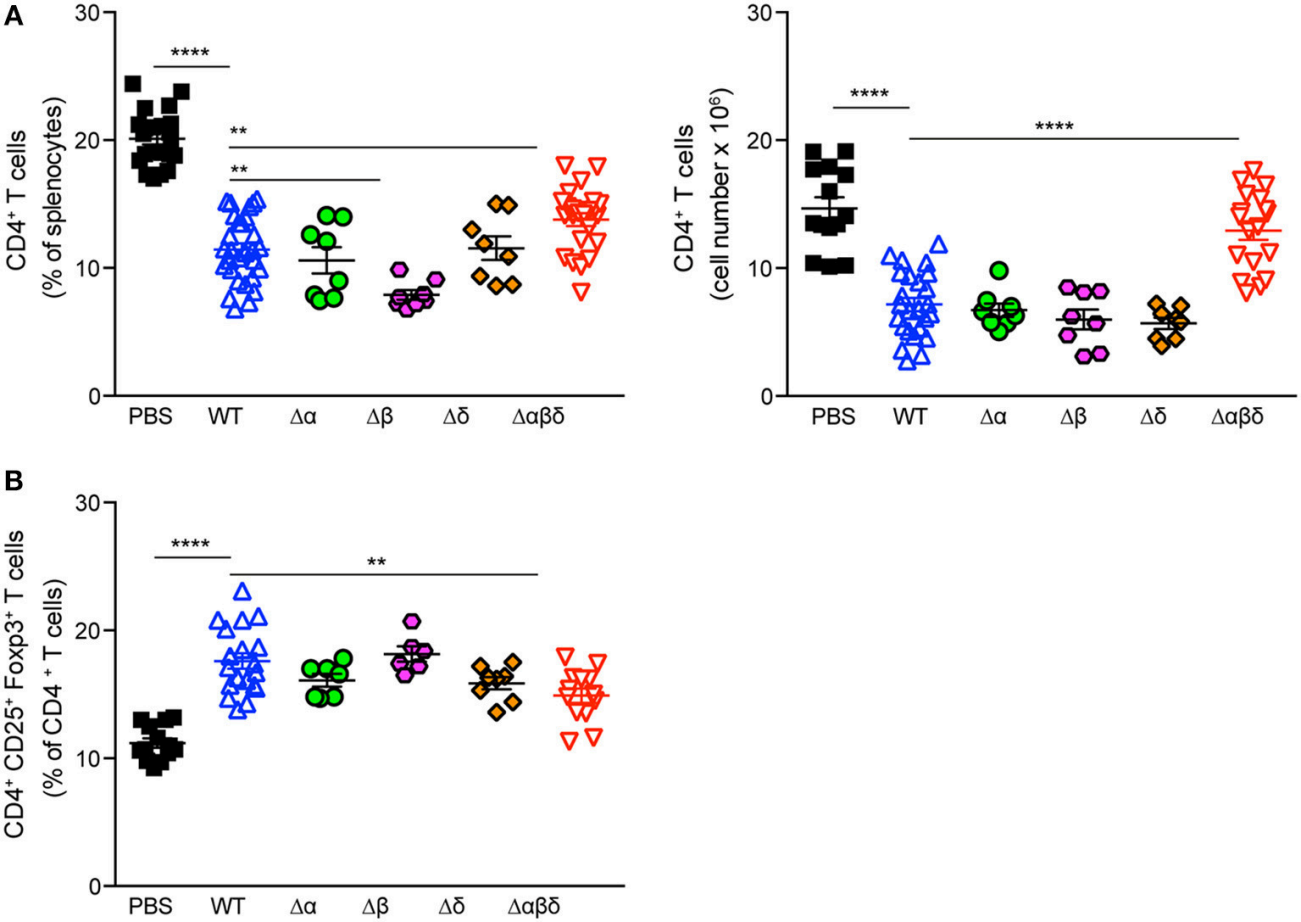
PSMα and δ peptides impair CD4^+^ T cell and promote T_reg_ responses. Foxp3-eGFP mice were either treated with PBS or infected with *S. aureus* USA300 WT or the indicated PSM-deletion mutant strains for 3 days and splenocytes were analyzed for CD4^+^ T cells by flow cytometry. Graphical summary of the frequency and cell numbers of splenic CD4^+^ T cells **(A)** and the frequencies of CD4^+^CD25^+^Foxp3^+^ T cells among CD4^+^ T cells **(B)**. Every symbol represents one mouse. The graphs represent the mean ± SEM with data pooled from multiple experiments; quantity of mice and experiments per condition and time is depicted in Table S2 (one-way ANOVA followed by Tukey's *post-hoc* test; differences are just shown compared to the USA300 WT; ^**^p < 0.01, ^****^p < 0.0001).

To address which PSM peptide is responsible for the *S. aureus* infection-induced reduction of CD4^+^ T cells and increased frequency of T_regs_, C57BL/6J or Foxp3-eGFP mice were infected with PSM deletion mutants, namely *S. aureus* USA300 Δα, *S. aureus* USA300 Δβ, or *S. aureus* USA300 Δδ. However, none of the single mutants behaved differently than the WT strain, suggesting a synergistic effect of the PSM peptides in impairing CD4^+^ T-cell and promoting T_reg_ responses (Figure 5).

### PSM-Dependent Inhibition of Th1 and Th17 Differentiation

**Figure 6:**
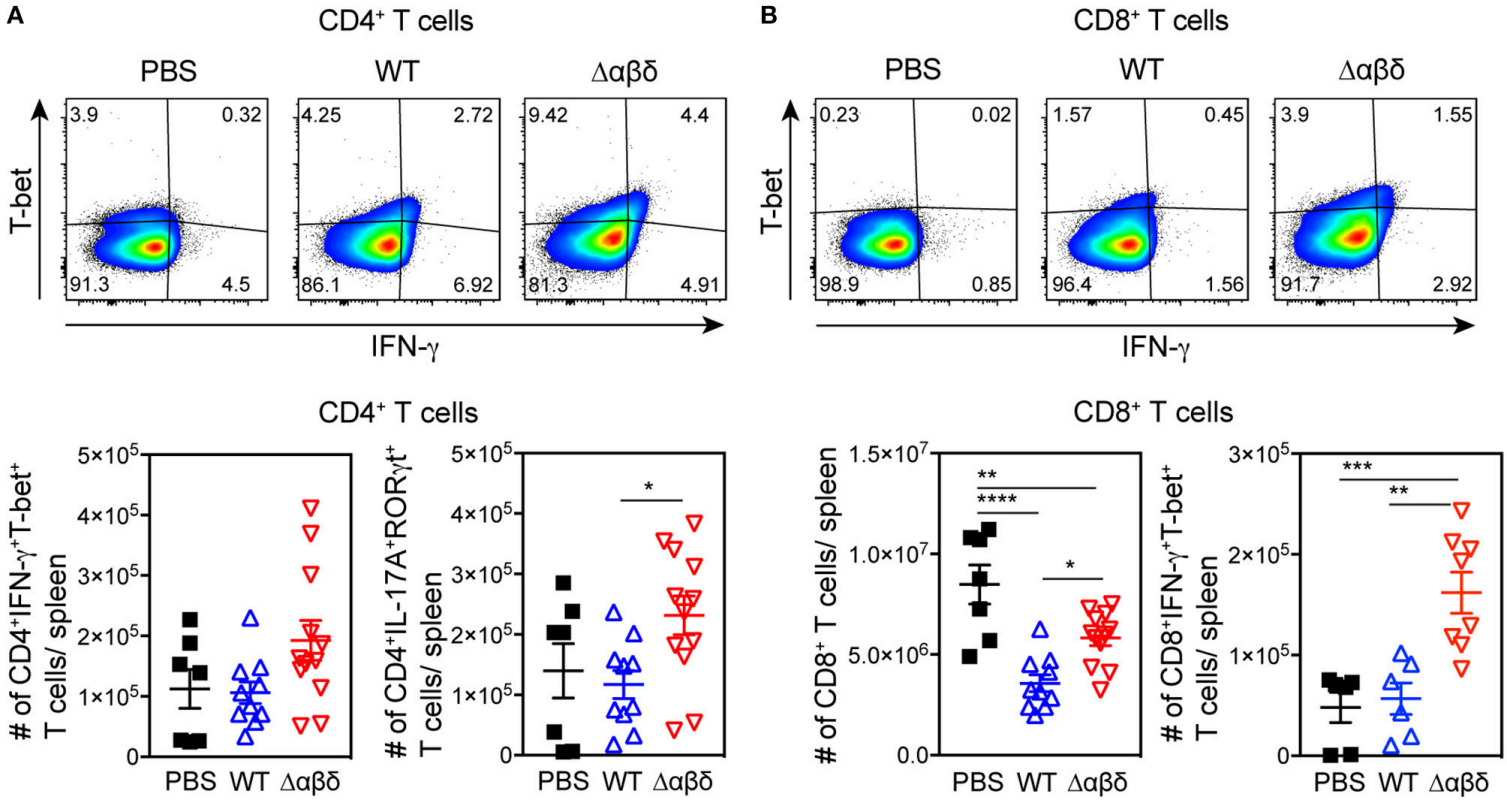
PSM-dependent inhibition of Th1 and Th17 polarization. C57BL/6J WT mice were either treated with PBS or infected with *S. aureus* USA300 WT or the PSM-deficient S. aureus USA300 Δαβδ mutant strain for 3 days and splenocytes were analyzed for CD4^+^ T cells by flow cytometry. **(A)** Pseudocolor plots show the frequency of IFN-γ^+^T-bet^+^ cells among CD4^+^ T cells in the spleen of PBS-treated or infected mice with the corresponding graphical summary of CD4^+^IFN-γ^+^T-bet^+^ and CD4^+^IL-17A^+^RORγt^+^ T cell numbers. **(B)** Pseudocolor plots show the frequency of IFN-γ ^+^T-bet^+^ cells among CD8^+^ T cells with the corresponding graphical summary of CD8^+^ and CD8^+^IFN-γ ^+^T-bet^+^ T cell numbers. Every symbol represents one mouse. The graphs represent the mean ± SEM with data pooled from multiple experiments; quantity of mice and experiments per condition and time is depicted in Table S2 (one-way ANOVA followed by Tukey's *post-hoc* test; ^*^p < 0.05, ^**^p < 0.01, ^***^p < 0.001, ^****^p < 0.0001).

To address whether PSMs have an effect on T-cell polarization *in vivo* CD4^+^ and CD8^+^ T cell subsets were analyzed *ex vivo* 72 h pi with *S. aureus* USA300 WT or USA300 Δαβδ mutant strain. The number of Th1 cells (gated as CD3^+^CD4^+^IFN-γ^+^T-bet^+^ cells) and Th17 cells (gated as CD3^+^CD4^+^IL-17A^+^RORγt^+^ cells) in the spleen of mice infected with the PSM deletion mutant *S. aureus* Δαβδ was twice as high compared to mice infected with the WT strain (Th1 cells: WT 1.06 ± 0.18 × 10^5^; Δαβδ mutant 1.93 ± 0.33 × 10^5^; Th17 cells: WT 1.17 ± 0.23 × 10^5^; Δαβδ mutant 2.31 ± 0.32 × 10^5^) (Figure 6A). Analyzing the effect of *S. aureus* infection on CD8^+^ T cells revealed a reduction of CD8^+^ T cell numbers in the spleen compared to PBS-treated mice, which was partially PSM-dependent (PBS: 8.48 ± 0.97 × 10^6^, WT: 3.57 ± 0.42 × 10^6^; Δαβδ mutant: 5.82 ± 0.38 × 10^6^) (Figure 6B). Despite this reduction, the numbers of CD8^+^IFN-γ^+^T-bet^+^ cells were similar in WT-infected and PBS-treated mice, but 3-fold increased in the spleen of mice infected with the PSM deletion mutant *S. aureus* Δαβδ (PBS: 0.48 ± 0.15 × 10^5^, WT: 0.56 ± 0.16 × 10^5^; Δαβδ mutant: 1.62 ± 0.2 × 10^5^). Moreover, overnight *in vitro* restimulation of splenocytes with PMA/ionomycin revealed similar results for IFN-γ^+^T-bet^+^CD4^+^ (Figure S8).

In summary, these data show that PSMs impair T-cell differentiation into IFN-γ-producing CD8^+^ T cells, Th1, and Th17 T cells upon *in vivo* infection with *S. aureus* USA300.

## Discussion

DCs sense the presence of staphylococcal components using PRRs like TLR2, NOD2, or FPR2. Clearance of *S. aureus* requires a protective immunity orchestrated by DCs (20). In the present study, we addressed the hypothesis that *S. aureus* PSMs disturb the adaptive immune response via modulation of DC subsets *in vivo*. We found that the numbers of DC subsets in the spleen, mainly CD4^+^ and CD8^+^ DCs, were reduced upon systemic infection with *S. aureus* USA300 independently of PSM secretion. Accordingly, other studies showed splenic DC-depletion in several bacterial infection models (27–31). Although the frequency of dead DCs was not increased throughout the infection, we cannot exclude that dead DCs were rapidly taken up by bystander cells (32). It is discussed that several mechanisms like apoptosis combined with impaired DC development are responsible for DC-depletion in systemic bacterial infection models (28, 31). Recently, it was shown that *S. aureus* leukocidin LukAB kills human DCs *in vitro* and in consequence inhibits activation and proliferation of primary human T cells (33). Moreover, LukED kills DCs, macrophages, and T cells by targeting CCR5 (34). Here, we showed significantly reduced the numbers of CD4^+^ and CD8^+^ T cells in the spleen upon *S. aureus* infection, which was even more pronounced for the PSM-secreting WT strain, indicating that PSMs in addition to leukocidins (35) mediate this cytolytic effect. However, it is still unclear how PSMs contribute to the reduced T cell count. Possibly directly via pore formation or indirectly via an FPR2-independent signaling cascade. Other cells than DCs and T cells also seem to be affected by *S. aureus* infection, which probably explains the here observed differences in frequency and numbers of CD4^+^ T cells (Figure 4B).

The maturation state of DCs and their cytokine profile upon pathogen encounter determines the outcome of T-cell responses (36). Here, we showed that CCR7 was upregulated on the surface of all DC subsets, indicating migration of these cells into the T-cell zones upon *S. aureus* infection (37). *S. aureus* infection induced the maturation of all DC subsets; PSMs had only minor enhancing effects. Besides, *S. aureus* infection elicited a strong pro-inflammatory cytokine response by splenocytes confirming recent findings (10), which was even enhanced by PSMs. These data are not consistent with *in vitro* studies performed with mouse bone marrow-derived DCs that showed complete inhibition of pro-inflammatory cytokine secretion upon treatment with TLR ligands and PSM α peptides (9, 21). These differences between the *in vitro* and *in vivo* studies could be explained by other splenocytes than DCs producing high amounts of pro-inflammatory cytokines. Other reasons could be lower amounts of PSMα peptides acting *in vivo* or due to antagonizing effects of other *S. aureus* pathogenicity factors, which were lacking in the *in vitro* studies. However, there was a trend of reduced TNF production at least by CD4^+^ and DN DC subsets upon infection with the PSM-secreting *S. aureus* USA300 WT strain. Moreover, PSMs specifically induced anti-inflammatory IL-10 production in the spleen. Similarly, *in vitro* studies showed that PSM-treated bone marrow-derived DCs produced high amounts of IL-10 and thereby increased the priming of T_regs_ and prevented that of Th1 cells (9, 11, 21). IL-10 was shown to play contrasting roles during *S. aureus* systemic and localized infections with prevention of bacterial dissemination and tissue damage due to T cell effector function or promotion of bacterial persistence by controlling T cell effector function, respectively (38). A large number of studies demonstrated that the induction of IL-10 secretion by various pathogens promoted T_reg_ development. These T_regs_ counteract T effector function, which prevents infection-induced immunopathology or prolongs pathogen persistence (39–41). Recent studies have highlighted the importance of T cell-mediated immune responses for *S. aureus* clearance (34). Thus, targeting and instructing DCs to become less stimulatory and in consequence prime regulatory responses would be a potent immune evasion strategy (42). The infection of mice with *S. aureus* led to a PSM-dependent increased frequency of T_regs_ in the spleen, which was independent of PSM recognition via the FPR2 receptor. Here, we additionally showed, that infection with various deletion mutants for PSMs act synergistically to increase T_regs_ *in vivo*. Concomitantly, Th1 and Th17 priming were impaired in a PSM-dependent manner upon *S. aureus* infection, suggesting a suppressive effect by T_regs_. These data confirm *in vitro* findings of PSM-treated human and mouse DCs favoring T_reg_ instead of Th1 or Th17 differentiation (9, 21, 22). The latter are protective against pathogens (43–47); thus this mode of action could be beneficial for *S. aureus* survival (5).

The relevance of PSMs in this infection model, however, seems to be limited as there is no dramatic phenotype of the mice infected with PSM secreting compared to PSM-deficient *S. aureus*. This lack could be because *S. aureus* USA300 is the most common cause of skin and soft tissue infections and not of bacteremia in humans. Also, the disease impact of PSMs secreted by *S. aureus* USA300 was demonstrated in a soft tissue infection model with swiss female mice and not C57BL/6 mice (5).

Together, our data support the notion that the modulation of DC subsets by PSMs into “less stimulatory” DCs promote T_reg_ responses in the spleen of the *S. aureus* USA300 WT infected mice. Due to findings by others demonstrating that splenic CD4^+^ DCs are superior for Th cell priming especially upon encounter of extracellular bacteria (12, 48) and the fact that PSMs impair TNF production by CD4^+^ DC subsets we speculate that these DCs are mainly responsible for T_reg_ priming upon *S. aureus* infection. Whether migratory or resident DCs induce T_reg_ priming remains to be determined. Moreover, it would be interesting to see whether the findings presented in this work are of relevance in other infection models reflecting, e.g., skin, soft tissue or respiratory diseases elicited by CA-MRSA strains.

## Ethics Statement

Animal experiments were performed in strict accordance with the German regulations of the Society for Laboratory Animal Science (GV-SOLAS) and the European Health Law of the Federation of Laboratory Animal Science Associations (FELASA). The protocol was approved by the Regierungspräsidium Tübingen (Permit Numbers: IZ2/12, M1/14).

## Author Contributions

NA, JR, and SA conceived the study, designed the experiments, and wrote the paper. NA, JR, JK, MG, and MB performed the experiments. JR, NA, MG, MB, and SA analyzed the data and SH proofread the statistical part of the paper. SA contributed reagents, materials, and analysis tools. All authors carefully read the manuscript.

### Conflict of Interest Statement

The authors declare that the research was conducted in the absence of any commercial or financial relationships that could be construed as a potential conflict of interest.

## Abbreviations

DC: dendritic cell
PSM: phenol-soluble modulin
TLR: Toll-like receptor
T_reg_: regulatory T cell
CA: community-associated
MRSA: methicillin-resistant *Staphylococcus aureus*
FPR2: formyl peptide receptor 2
PRR: pattern recognition receptors
TLR, Toll-like receptor, WT: wild type.
