# Supplementary material for: PSM Peptides From Community-Associated Methicillin-Resistant Staphylococcus aureus Impair the Adaptive Immune Response via Modulation of Dendritic Cell Subsets in vivo
Source: Front Immunol. 2019 May 10;10:995. doi: 10.3389/fimmu.2019.00995 (PMC6524657; doi:10.3389/fimmu.2019.00995)
Supplement: Supplementary file 1 [file Data_Sheet_1.pdf]

PSM peptides from community-associated methicillin-resistant *Staphylococcus aureus* impair the adaptive immune response via modulation of dendritic cell subsets *in vivo*

Jennifer R. Richardson[1*], Nicole S. Armbruster[1*], Manina Günter[1], Michelle Biljecki[1], Juliane Klenk[1], Simon Heumos[2], and Stella E. Autenrieth[1]

[1]Department of Internal Medicine II, University of Tübingen, Tübingen, Germany
[2]Quantitative Biology Center (QBiC), University of Tübingen, Tübingen, Germany

* contributed equally

Supplement Material

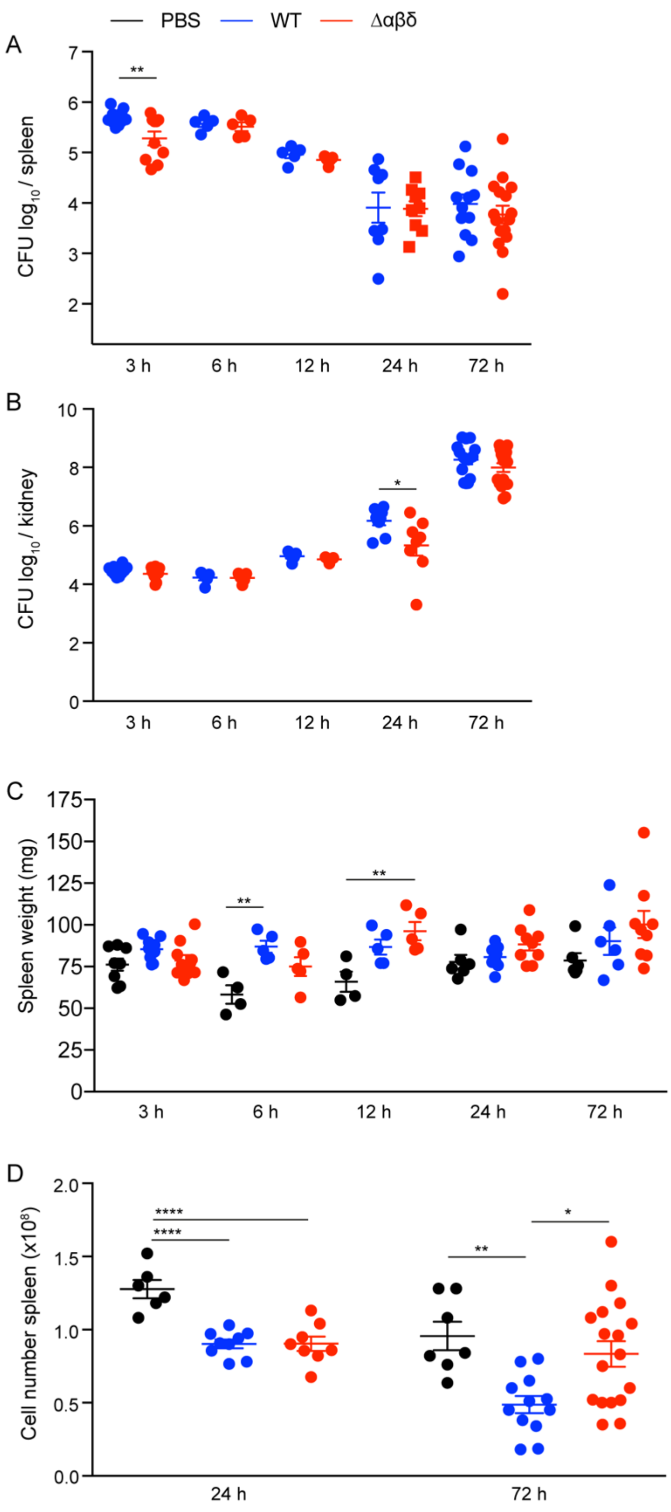

Figure S1: Bacterial load of spleen and kidney, spleen weight and cell numbers

C57BL/6 WT mice were either treated with PBS or infected with *S. aureus* USA300 WT or the PSM-deficient *S. aureus* USA300 Δαβδ mutant strain for up to 72 h. The bacterial load of the mice infected with *S. aureus* USA300 or *S. aureus* USA300 Δαβδ was assessed in (A) the spleen and in (B) the kidney by plating serial dilutions on agar

plates. Graphical summary of the CFU per (A) spleen and (B) kidney over time starting at the detection limit of 1.2. (Unpaired student's T-test; $p < 0.05$ (*), $p < 0.01$ (**)). (C) Graphical summary of the spleen weight of PBS-treated or infected mice over time. (D) Graphical summary of the cell numbers of the spleen of PBS-treated or infected mice after 24 h and 72 h of infection. (one-way ANOVA followed by Tukey's post-hoc test; $p < 0.05$ (*), $p < 0.01$ (**), $p < 0.0001$ (****)). Every symbol represents one mouse. The graphs represent the mean ± SEM with data pooled from multiple experiments; quantity of mice and experiments per condition and time see supplement table 2.

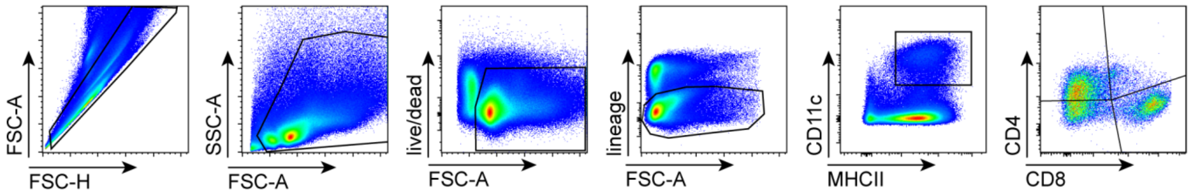

Figure S2: Gating strategy of DCs and DC subsets in the spleen

C57BL/6 WT, FPR2$^{-/-}$, or Foxp3-eGFP mice were either treated with PBS or infected with *S. aureus* USA300 WT or the PSM-deficient *S. aureus* USA300 Δαβδ mutant strain for up to 72 h and splenocytes were analyzed for DC subsets by flow cytometry. Splenic DCs were characterized as singlets, leukocytes, living, lineage (CD19, NK1.1, Ter-119, GR-1, CD90.2)$^{-}$, CD11c^{+}MHCII^{+} cells and subdivided into the DC subsets by their expression of CD4 and CD8.

Figure S3

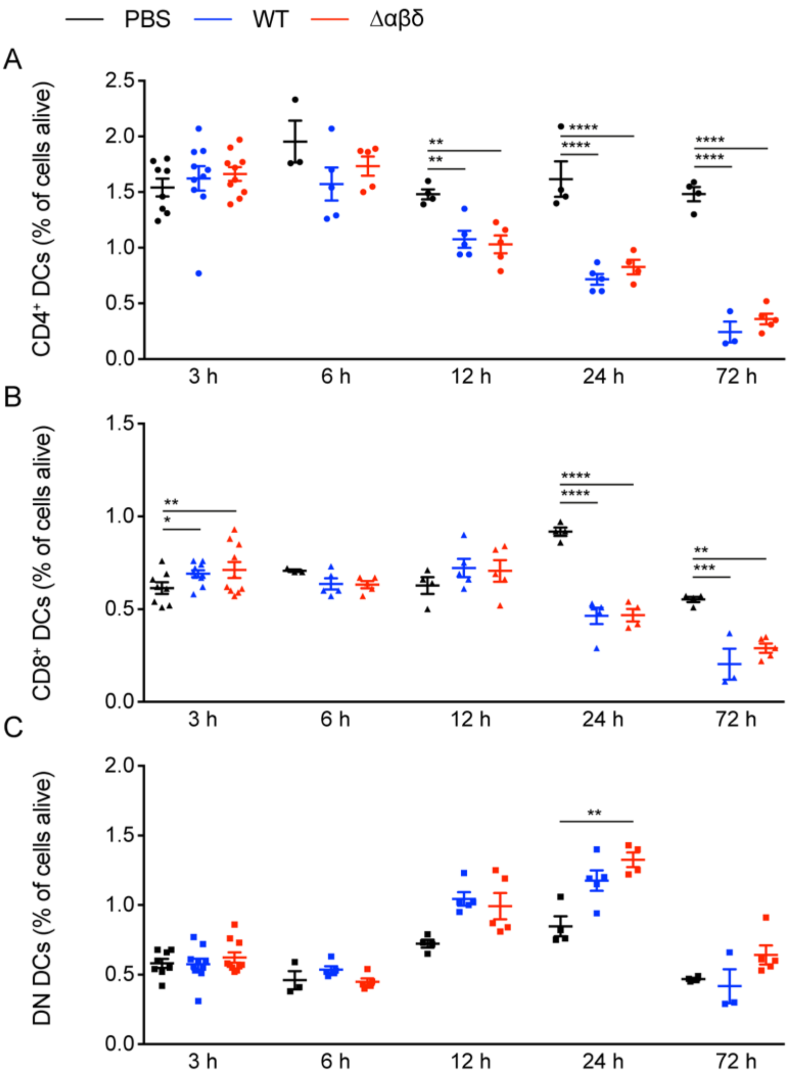

Figure S3: Reduced frequencies of splenic DC subsets upon *S. aureus* infection

C57BL/6 WT mice were either treated with PBS or infected with *S. aureus* USA300 WT or the PSM-deficient *S. aureus* USA300 Δαβδ mutant strain for up to 72 h and splenocytes were analyzed for the different DC subsets by flow cytometry. Graphical summary of the frequencies of splenic (A) $CD4^+$, (B) $CD8^+$ and (C) DN DCs over time. Every symbol represents one mouse. The graphs represent the mean ± SEM with data pooled from multiple experiments; quantity of mice and experiments per condition and time see supplement table 2 (one-way ANOVA followed by Tukey's post-hoc test; $p < 0.05$ (*), $p < 0.01$ (**), $p < 0.001$ (***), $p < 0.0001$ (****)).

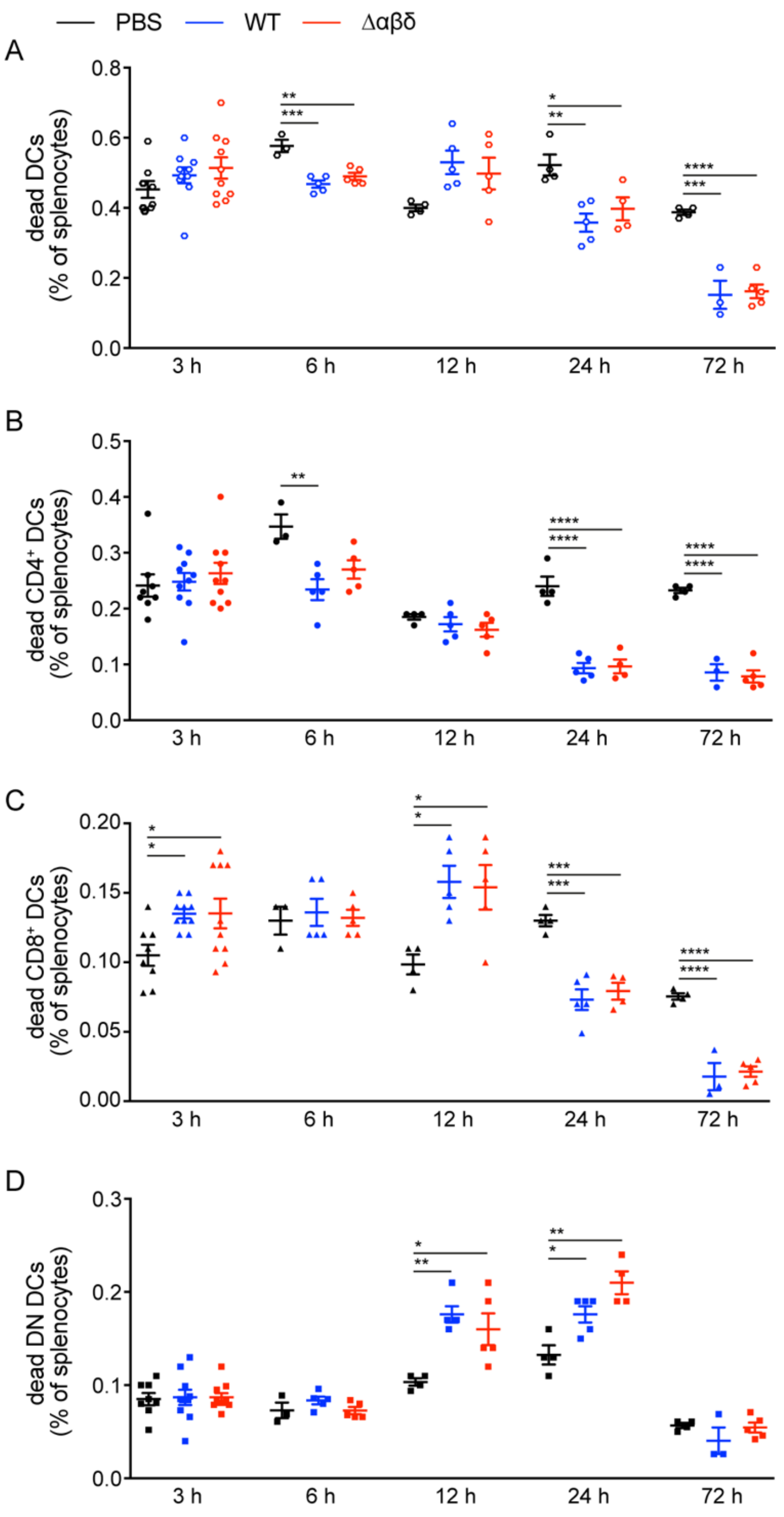

Figure S4:

C57BL/6 WT mice were either treated with PBS or infected with *S. aureus* USA300 WT or the PSM-deficient *S. aureus* USA300 Δαβδ mutant strain for up to 72 h and

splenocytes were analyzed for dead total DCs and dead DC subsets by flow cytometry. Graphical summary of the frequencies of dead (A) total DCs, (B) $CD4^+$, (C) $CD8^+$ and (D) DN DCs over time. Every symbol represents one mouse. The graphs represent the mean ± SEM with data pooled from multiple experiments; quantity of mice and experiments per condition and time see supplement table 2 (one-way ANOVA followed by Tukey's post-hoc test; $p < 0.05$ (*), $p < 0.01$ (**), $p < 0.001$ (***), $p < 0.0001$ (****)).

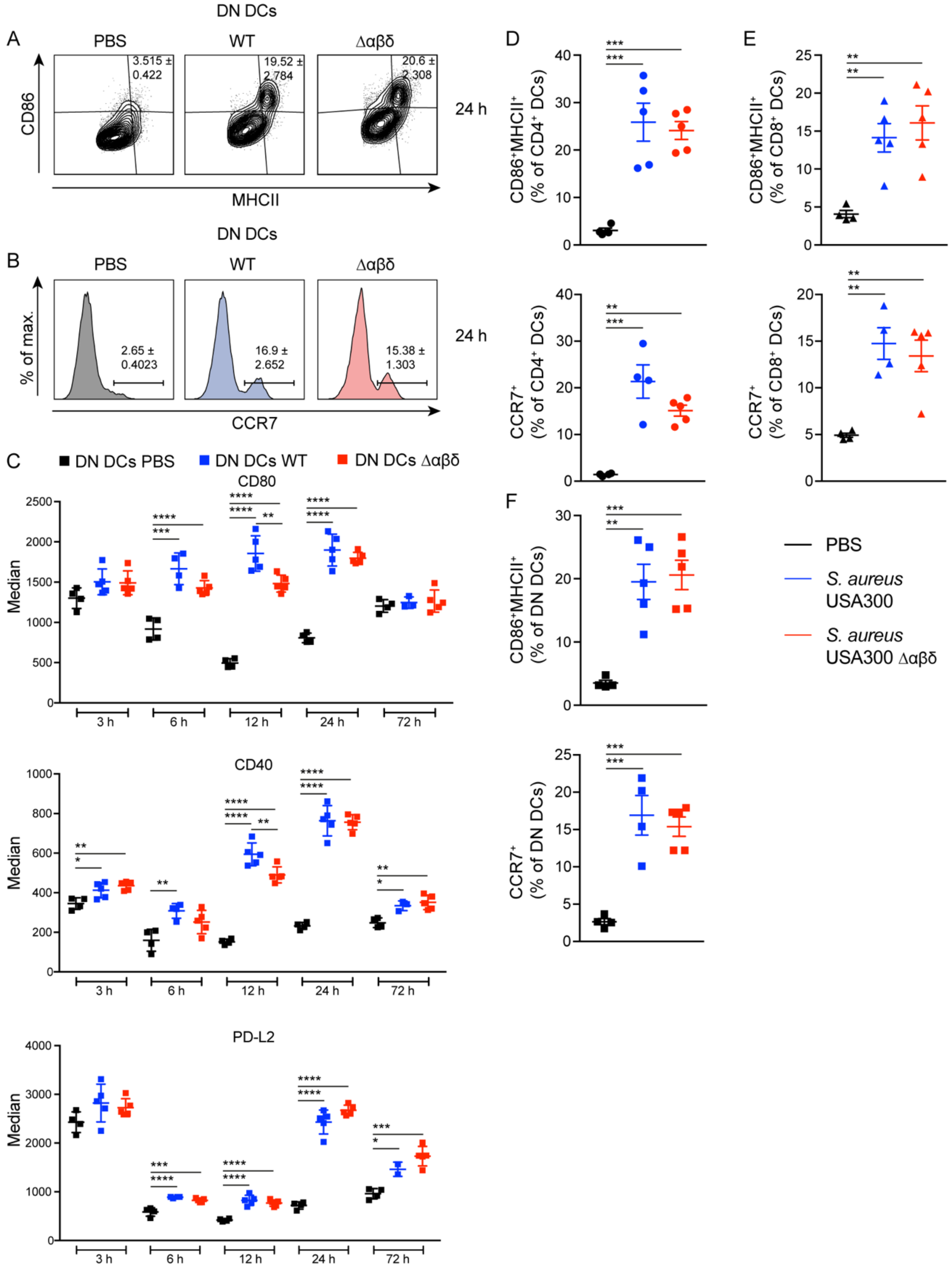

Figure S5: *S. aureus* infections induce DC maturation

C57BL/6 WT mice were either treated with PBS or infected with *S. aureus* USA300 WT or the PSM-deficient *S. aureus* USA300 Δαβδ mutant strain for up to 72 h and splenocytes were stained with CD11c, MHC class II, CCR7, CD80, CD86, CD40 and PD-L2 antibodies and analyzed by flow cytometry. Representative contour plots and

histograms show the frequency of (A) CD86$^+$MHCII$^+$ cells and (B) CCR7$^+$ cells, respectively, among DN DCs in the spleen of PBS-treated or infected mice. (C) Graphical summary of the median expression of CD80, CD40 and PD-L2 by DN DCs in the spleen of PBS-treated or infected mice over time. Data represent the mean ± SEM (one-way ANOVA followed by Tukey's post-hoc test; $p < 0.05$ (*), $p < 0.01$ (**), $p < 0.001$ (***), $p < 0.0001$ (****)). (D-F) Graphical summary of the frequency of CD86$^+$MHCII$^+$ cells and CCR7$^+$ cells among (D) CD4$^+$ DCs, (E) CD8$^+$ DCs, and (F) DN DCs. Data represent the mean ± SEM (one-way ANOVA followed by Tukey's post-hoc test; $p < 0.01$ (**), $p < 0.001$ (***)). Every symbol represents one mouse; quantity of mice and experiments per condition and time see supplement table 2.

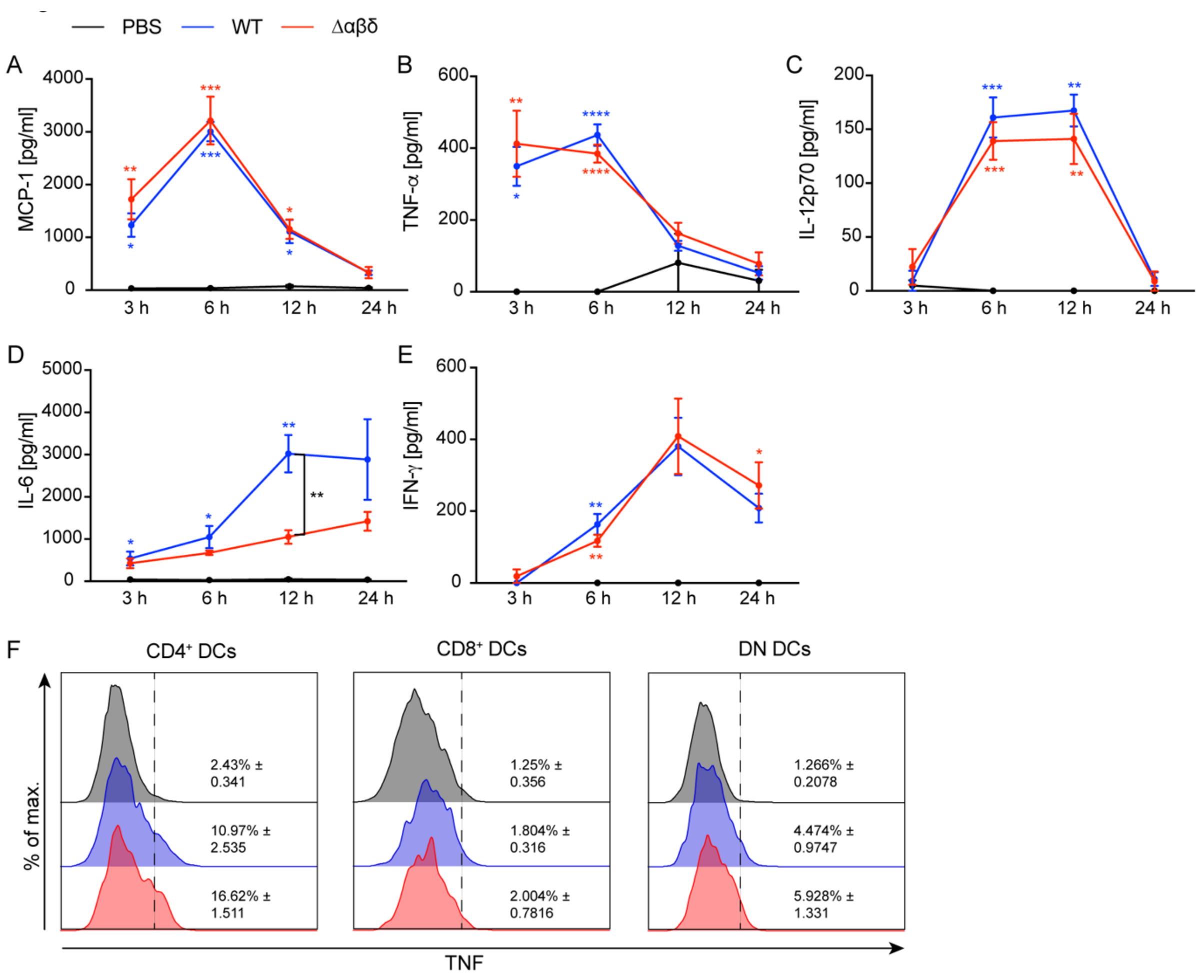

Figure S6: *S. aureus* PSMs modulate the pro-inflammatory and anti-inflammatory cytokine response upon *in vivo* infection

C57BL/6 WT mice were either treated with PBS or infected with *S. aureus* USA300 WT or the PSM-deficient *S. aureus* USA300 Δαβδ mutant strain for up to 24 h. At the indicated times blood plasma was analyzed for (A) MCP-1, (B) TNF, (C) IL-12p70, (D) IL-6, or (E) IFN-γ by multiplex bead array. The graphs represent the mean ± SEM with data pooled from multiple experiments; quantity of mice and experiments per condition and time is depicted in supplementary table 2 (one-way ANOVA followed by Tukey's post-hoc test; $p < 0.05$ (*), $p < 0.01$ (**), $p < 0.001$ (***), and $p < 0.0001$ (****); blue * describe differences between PBS and *S. aureus* USA300 WT, red * describe differences between PBS and *S. aureus* USA300 Δαβδ, and black * describe differences between *S. aureus* USA300 WT and USA300 Δαβδ. (F) Histogram overlay

of TNF^+ cells among DC subsets in the spleen of PBS-treated or infected mice. One representative from each treatment out of one experiment with 5 mice per condition.

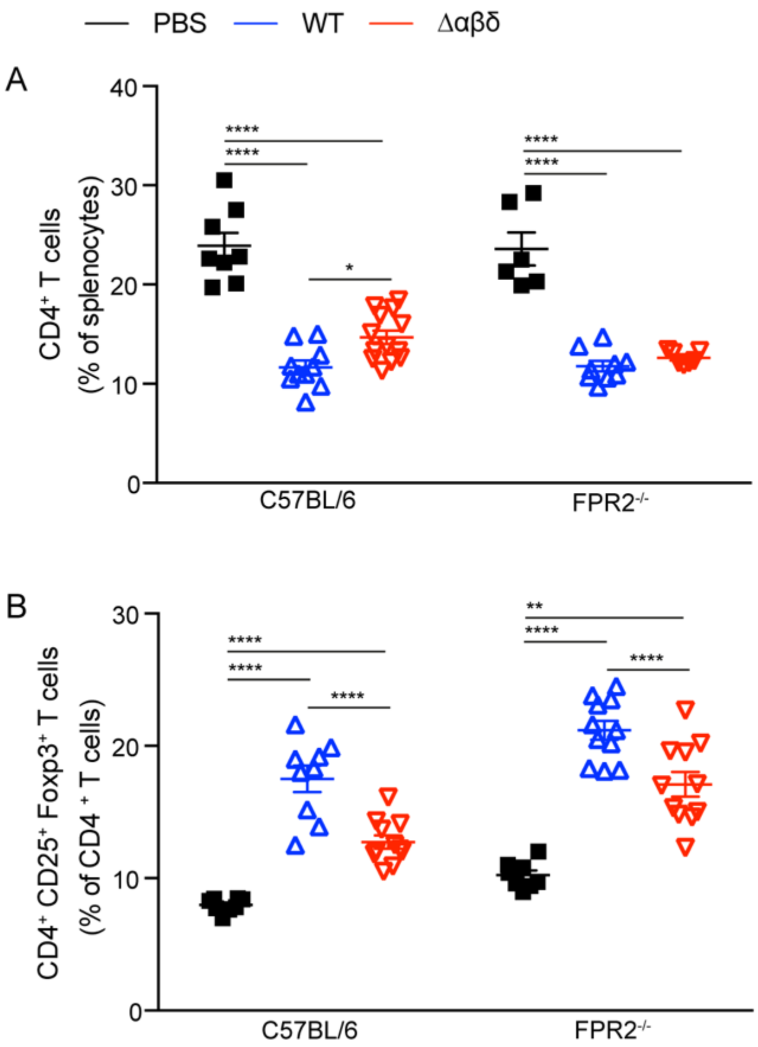

Figure S7: The by PSM-altered T cell response is FPR2-independent

C57BL/6 WT or FPR2$^{-/-}$ mice were either treated with PBS or infected with *S. aureus* USA300 WT or the PSM-deficient *S. aureus* USA300 Δαβδ mutant strain for 72 h and splenocytes were analyzed for CD4^{+} T cells by flow cytometry. Graphical summary of the (A) frequencies of splenic CD4^{+} T cells, and of the (B) frequencies of CD4^{+}CD25^{+}FoxP3^{+} T cells. Every symbol represents one mouse. The graphs represent the mean ± SEM with data pooled from multiple experiments; quantity of mice and experiments per condition and time see supplement table 2 (one-way ANOVA followed by Tukey's post-hoc test; $p < 0.05$ (*), $p < 0.0001$ (****)).

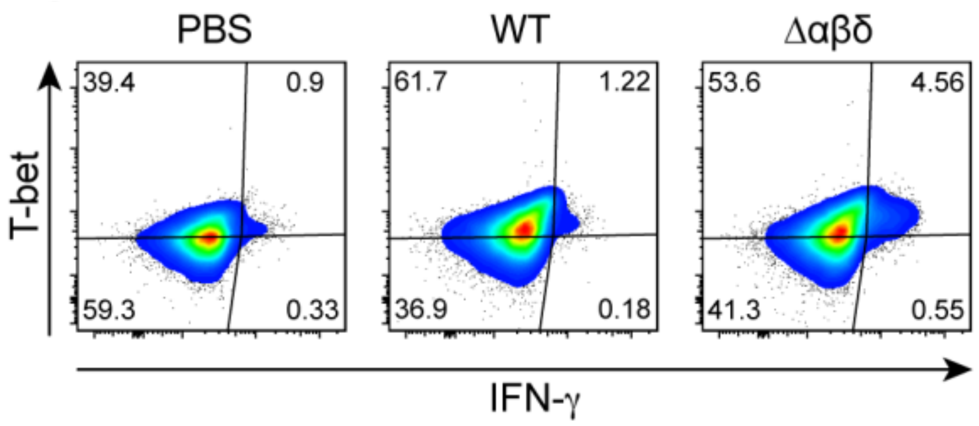

Figure S8: The presence of PSM peptides reduced the infection induced IFN-γ secretion

C57BL/6 WT mice were either treated with PBS or infected with *S. aureus* USA300 WT strain or the PSM-deficient S. aureus USA300 Δαβδ mutant strain for 72 h. Splenocytes were harvested and restimulated with PMA/ionomycin for 6 h followed by flow cytometry staining. $CD4^{+}IFN\text{-}\gamma^{+}T\text{-}bet^{+}$ T cells were analyzed by flow cytometry. Representative pseudocolor plots show the frequency of $IFN\text{-}\gamma^{+}T\text{-}bet^{+}$ cells among $CD4^{+}$ T cells in the spleen of PBS-treated or infected mice.

Table 1: Antibody panels used in the study

Panel	Mice	Antigen	Fluorochrome	Clone	Company
T_{reg} Panel	FoxP3-eGFP	CD3ε	APC-Cy7	145-2C11	BioLegend
		CD4	eFluor450	RM4-5	eBioscience
		CD25	APC	PC61	BioLegend
	C57BL/6/ $FPR2^{-/-}$	CD3ε	APC-Cy7	145-2C11	BioLegend
		CD4	eFluor450	RM4-5	eBioscience
		CD25	APC	PC61	BioLegend
		FoxP3	PE	FJK-16s	eBioscience

Panel	Mice	Antigen	Fluorochrome	Clone	Company
DC Panel	C57BL/6	CD4	BV510	GK1.5	BioLegend
		CD8α	BV785	53-6.7	BioLegend
		CD11c	APC	N418	Miltenyi
		CD40	PerCP-Cy5.5	3/23	BioLegend
		CD80	BV650	16-10A1	BioLegend
		CD86	BV605	GL-1	BioLegend
		CCR7	BV421	4B12	BioLegend
		MHCII	FITC	M5/114.15.2	Miltenyi
		PD-L2	PE-Dazzle	10F.9G2	BioLegend
		CD19 (lin)	AlexaFluor700	6D5	BioLegend
		CD90.2 (lin)	AlexaFluor700	30-H12	BioLegend
		NK1.1 (lin)	AlexaFluor700	PK136	BioLegend
		Ter-119 (lin)	AlexaFluor700	Ter-119	BioLegend
		GR-1 (lin)	AlexaFluor700	RB6-8C5	BioLegend

Panel	Mice	Antigen	Fluorochrome	Clone	Company
T cell panel	C57BL/6	CD3ε	PerCP-Cy5.5	145-2C11	BioLegend
		CD4	BV510	GK1.5	BioLegend
		CD8α	BV785	53-6.7	BioLegend
		CD25	PE-Cy7	PC61	BioLegend
		CD11b (lin)	AlexaFluor700	M1/70	BioLegend
		CD11c (lin)	AlexaFluor700	N418	BioLegend
		CD19 (lin)	AlexaFluor700	6D5	BioLegend
		CD90.2 (lin)	AlexaFluor700	30-H12	BioLegend
		NK1.1 (lin)	AlexaFluor700	PK136	BioLegend
		Ter-119 (lin)	AlexaFluor700	Ter-119	BioLegend
		Gr-1 (lin)	AlexaFluor700	RB6-8C5	BioLegend
		FoxP3	APC	FJK-16s	eBioscience
		IFN-γ	BV650	XMG1.2	BioLegend
		IL-10	PE	JES5-16E3	Miltenyi
		IL-17A	BV421	TC11-18H10	BioLegend
		RORγt	PE-eFluor610	B2D	eBioscience
		T-bet	FITC	4B10	BioLegend

Table 2: Quantity of mice and experiments per condition and time used in this study

Figure	Time	PBS	USA300 WT	USA300 Δαβδ	USA300Δα or Δβ or Δδ	# experiments
1 + S3	3 h	8	10	10		2
	6 h	4	5	5		1
	12 h	4	5	5		1
	24 h	9/ 11 (total DCs)	10/ 14 (total DCs)	10/ 14 (total DCs)		2/ 3 (total DCs)
	72 h	7/ 8 (total DCs)	10/ 14 (total DCs)	13/ 17 (total DCs)		3/ 4 (total DCs)
2 + S5	3 h	4	5	5		2
	6 h	4	4	5		1
	12 h	4	5	5		1
	24 h	4	5	5		2
	72 h	4	2	5		1
3 A-I	3 h	6	9	10		2
	6 h	3	5	5		1
	12 h	4	5	5		1
	24 h	2	5	5		2
3 J + S6F		5	5	5		1
4 B		23	31	25		6-10
4 C		13	20	13		3-8
5		23	31	25	8	2-11
6		7	10	12		3
S1 A	3 h	-	10	10		2
	6 h	-	5	5		1
	12 h	-	5	5		1
	24 h	-	9	8		2
	72 h	-	12	16		4
S1 B	3 h	-	10	10		2
	6 h	-	5	5		1
	12 h	-	5	5		1
	24 h	-	9	8		2
	72 h	-	14	17		4
S1 C, D	3 h	8	9	10		2
	6 h	4	5	5		1
	12 h	4	5	5		1
	24 h	6	9	9		2
	72 h	6	6	9		2
S4	3 h	8	10	10		2
	6 h	3	5	5		1
	12 h	4	5	5		1
	24 h	4	5	4		2
	72 h	4	3	5		1
S6 A-E	3 h	4	4	5		2
	6 h	3	5	5		1
	12 h	2	5	5		1
	24 h	2	4	4		2
S7	C57BL/6	8	10	12		4
	$FPR2^{-/-}$	8	11	11		4
